# Bilateral breast nodules as an unusual manifestation of 17α-hydroxylase/17,20-lyase deficiency

**DOI:** 10.3389/fendo.2025.1658362

**Published:** 2025-10-27

**Authors:** Minchun Zhang, Xing Huang, Qifeng Li, Shuyan Gui, Shiwei Lin, Gang Fan, Jing Yang

**Affiliations:** ^1^ Department of Endocrinology, Shenzhen Nanshan People’s Hospital, Affiliated Nanshan Hospital of Shenzhen University, Shenzhen, China; ^2^ Guangdong Key Laboratory for Biomedical Measurements and Ultrasound Imaging, National-Regional Key Technology Engineering Laboratory for Medical Ultrasound, School of Biomedical Engineering, Shenzhen University Medical School, Shenzhen, China; ^3^ Pan-Vascular Research Group, Shenzhen Nanshan People’s Hospital, Affiliated Nanshan Hospital of Shenzhen University, Shenzhen, China; ^4^ Department of Pathology, Shenzhen Nanshan People’s Hospital, Affiliated Nanshan Hospital of Shenzhen University, Shenzhen, China; ^5^ Department of Radiology, Shenzhen Nanshan People’s Hospital, Affiliated Nanshan Hospital of Shenzhen University, Shenzhen, China; ^6^ Medical Research Center, Shenzhen Nanshan People’s Hospital, Affiliated Nanshan Hospital of Shenzhen University, Shenzhen, China

**Keywords:** 17α-hydroxylase/17,20-lyase deficiency, breast nodules, mammary duct ectasia, *CYP17A1*, breast development

## Abstract

**Introduction:**

17α-hydroxylase/17,20-lyase deficiency (17-OHD) typically presents with sexual infantilism, hypertension, and hypokalemia. However, phenotypic variability, particularly breast development, may obscure diagnosis. This study aims to characterize an atypical presentation of 17-OHD with preserved breast development and breast nodules, and to evaluate clinical and hormonal features associated with breast development through a systematic literature review.

**Methods:**

A 38-year-old woman with bilateral breast nodules and ductal ectasia was diagnosed with 17-OHD, confirmed by *CYP17A1* variants. A literature review of 17-OHD cases with near-complete breast development (Tanner stage 4–5) was conducted to analyze clinical, hormonal, and genotypic features.

**Results:**

The patient exhibited classic signs of 17-OHD including hypertension, hypokalemia, adrenal hyperplasia, and hypogonadism, but also presented with atypical bilateral breast nodules and mammary duct ectasia. Hormone therapy resulted in clinical improvement and regression of the breast findings. Literature analysis of 43 patients with breast development showed that patients with 46,XX were diagnosed later than 46,XY (29.5 ± 11.5 vs. 19.8 ± 6.9 years, *P* = 0.0095). Estradiol was more often subnormal in 46,XX, while both groups showed progesterone excess and androgen deficiency. Pubic hair development differed by karyotype (*P* = 0.027), which was more advanced in the 46,XY group. Genetic data revealed that breast development was associated with non-null *CYP17A1* variants, and most variants clustered in exons 5–8, with exon 8 as a hotspot.

**Conclusion:**

This case broadens the phenotypic spectrum of 17-OHD, highlighting that preserved breast development and benign breast lesions may delay diagnosis. Literature review suggests partial loss-of-function variants contribute to this phenotype. Greater awareness is essential to prevent misdiagnosis and unnecessary interventions.

## Introduction

17α-hydroxylase/17,20-lyase deficiency (17-OHD) is a rare autosomal recessive disorder caused by variants in the *CYP17A1* gene, accounting for approximately 1% of congenital adrenal hyperplasia (CAH) cases (1, 2). The enzyme plays a pivotal role in steroidogenesis by catalyzing two reactions: 17α-hydroxylase activity which is essential for cortisol production, and 17,20-lyase activity for generating sex hormone precursors (dehydroepiandrosterone and androstenedione). Consequently, the deficiency leads to a characteristic clinical trial: (1) hypocortisolemia (84.5%) due to impaired cortisol synthesis, (2) hypergonadotropic hypogonadism with disordered sexual development (59.5%) from sex steroid deficiency, and (3) hypertension (57%) with hypokalemia (45.4%) resulting from mineralocorticoid excess (3).

Clinically, 17-OHD manifests as a spectrum of disease severity, which is classified into complete and partial forms based on residual enzymatic activity (1). While the classic manifestations are well-recognized, emerging evidence reveals that there are some atypical features of the 17-OHD, including short stature due to the absence of a pubertal growth spurt during adolescence (4), the development of testicular tumors (5), and malignant mixed germ cell tumors (6). This phenotypic variability often leads to diagnostic delays or misdiagnosis, emphasizing the need for clinicians to recognize the broad clinical spectrum of 17-OHD.

To date, adrenal hyperplasia and ovarian cysts remain the most documented imaging findings in 17-OHD (7, 8). While breast underdevelopment is widely reported, 64.2% of patients exhibit some degree of breast development, and only 4.3% demonstrate near-complete to complete development (3). In this study, we describe bilateral breast nodules as an unusual manifestation in a patient with 17-OHD, which showed near-complete resolution following hormonal therapy. Additionally, we conducted a systematic review of previously reported 17-OHD cases presenting with nearly complete breast development. These findings underscore the importance of considering steroidogenic disorders in the differential diagnosis of breast pathology, potentially preventing unnecessary surgical interventions through early endocrine evaluation.

## Methods

### Participants

The study was approved by the Ethics Committee of Shenzhen Nanshan People’s Hospital. Written informed consent was obtained from the patient to share the data including demographic characteristics, clinical presentations, laboratory tests, radiological and pathological findings. All research procedures complied with the Declaration of Helsinki guidelines.

### Measurement of plasma adrenal steroid hormones

The measurement of steroid hormones was performed using ultra-performance liquid chromatography coupled with tandem mass spectrometry (UPLC-MS/MS) on a Waters Xevo TQ-S IVD system in a commercial laboratory, KingMed Diagnostics (https://en.kingmed.com.cn/).

### Genetic test

Genomic DNA was isolated from peripheral blood sample of the patient using QIAamp DNA Blood Mini kit (Qiagen, Dusseldorf, Germany). The karyotyping and whole-exome exoneme sequencing were performed in a commercial laboratory, KingMed Diagnostics following established protocols (9).

### Literature review and data analysis

To systematically evaluate breast phenotype in patients with 17-OHD, we initially reviewed 176 studies included in a recent systematic review (covering literature up to 2022) (3). An updated PubMed search using the term “*CYP17A1* deficiency” was performed (through April 2025), identifying 7 additional cases. Two physicians independently screened all studies and extracted data on patients with breast development (Tanner stage B4–5) where reported.

The following parameters were extracted: age, karyotype, phenotypic sex, height, weight, body mass index (BMI), blood pressure, serum potassium levels, pubertal development stage, hormonal profiles, and genetic variants. Given the variability in laboratory reference ranges across studies, hormone levels were categorized as subnormal, normal, or elevated relative to each study’s reported reference values.

Genetic variants were systematically classified as either null or non-null based on their predicted impact on the protein product according to the American College of Medical Genetics and Genomics guidelines and established literature (3). Null variants were defined as those expected to completely abolish enzymatic function (e.g., nonsense, frameshift, initiation codon variants, single or multi-exon deletions, and canonical splice site variants at positions ±1 or 2). Non-null variants were defined as those likely to reduce but not entirely eliminate enzymatic function including missense variants and in-frame deletions/insertions. These cases were categorized as C2 (two non-null variants) or NC (one null and one non-null variant).

Statistical comparisons of clinical parameters were conducted using Student’s t-test for continuous variables and the χ² test (Chi-square test) for categorical variables to evaluate differences between karyotype groups (46,XX vs. 46,XY) and genotype-based subgroups (C2 vs. NC).

## Results

### Clinical management during hospitalization

**Figure 1:**
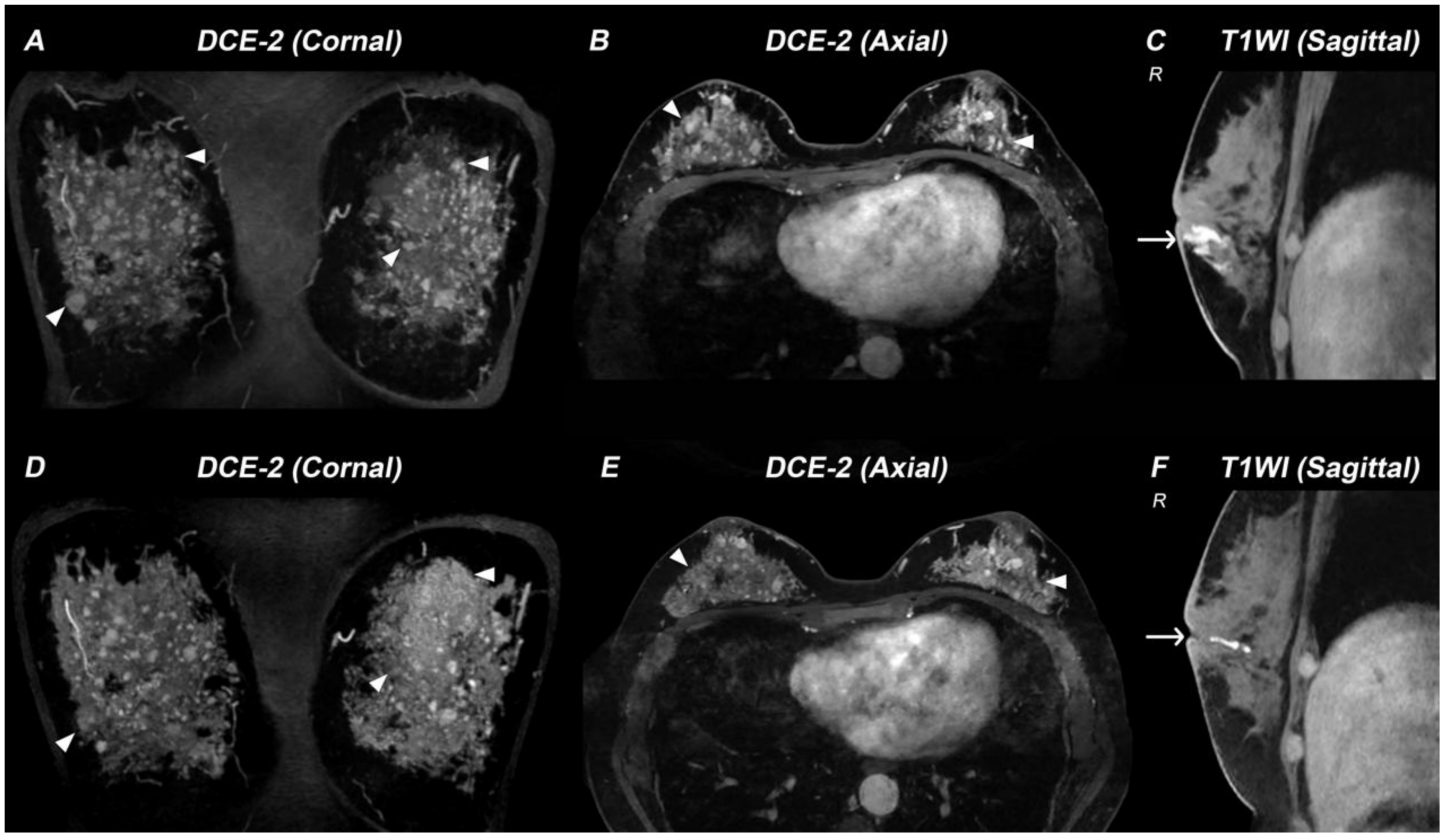
Breast MRI demonstrating therapy-related changes in a patient with 17α-hydroxylase/17,20-lyase deficiency (17-OHD). **(A–C)** MR images acquired before 17-OHD therapy: DCE-2 MRI **(A, B)** images and T1WI image **(C)** show multiple nodules (triangle) and enlarged mammary ducts (arrow) in the breasts of this case. **(D–F)** MR images acquired six months after 17-OHD therapy: DCE-2 MRI **(D, E)** and T1WI image **(F)** show significant reduction of nodules (triangle) and mammary ducts (arrow).

A 38-year-old woman (46,XX) presented with a 12-year history of bilateral breast nodules, having undergone two previous excisional biopsies that revealed intraductal papilloma and mammary duct ectasia (**Supplementary Figure S1**). Pre-admission MRI revealed mammary duct ectasia and multiple ring-enhancing nodules of varying sizes in both breasts (**Figures 1A–C**). Upon admission, she had hypertension (195/135 mmHg) and hypokalemia (2.73 mmol/L). Physical examination showed a height of 159 cm, weight of 54 Kg, BMI of 21.4 Kg/m² and tanner B4P1 development (**Supplementary Figure S1**). Her menstrual cycles began at age 14 and remained regular, though with consistently scant flow.

**Figure 2:**
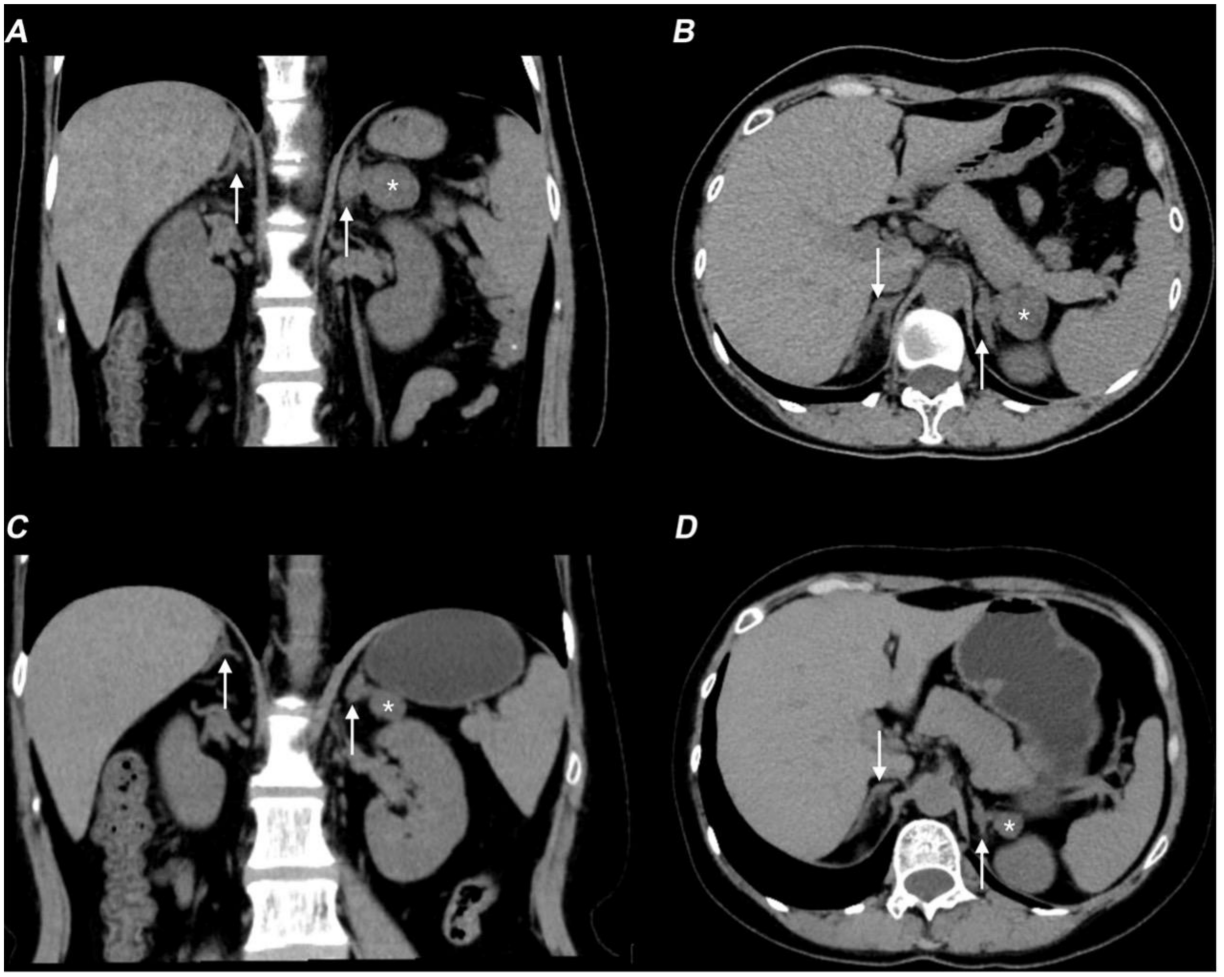
Non-contrast CT scan showing therapy-related adrenal changes in the patient with 17-OHD. **(A, B)** Axial **(A)** and coronal **(B)** non-contrast CT images show bilaterally enlarged adrenal glands (arrows) and a lesion (*) in the adrenal gland before therapy. **(C, D)** Axial **(C)** and coronal **(D)** non-contrast CT images show improvement in bilateral adrenal gland thickening (arrows) and a decrease in the size of the left adrenal mass (*).

Hormonal profiling (**Table 1**) showed hypogonadism (low estradiol [21 ng/L] and testosterone [<0.10 ng/mL]), with elevated gonadotropins (FSH 11.36 mIU/mL, LH 17.96 mIU/mL), and adrenal insufficiency (cortisol 79 nmol/L with ACTH elevation [96.3 pg/mL]). She also exhibited hypokalemia, low aldosterone levels, and decreased renin activity. CT showed a left adrenal mass and right adrenal hypertrophy (**Figures 2A, B**), while ultrasound detected enlarged multi-cystic ovaries.

**Table 1:** Laboratory data before and six months after therapy for the patient with 17-OHD.

Laboratory Test	Before therapy	Six months after therapy	Typical range
Serum Potassium, mmol/L	2·73	4·19	3·5-5·3
Cortisol, nmol/L	79·0	51·5	Morning: 185-624
Adrenocorticotropic hormone, pg/mL	96·3	61·4	Morning: 7·2-63·3
Aldosterone, pg/mL	67·8	248·7	40-310
Renin activity, ng/mL/hr	2·64	17·51	1·31-3·95
Aldosterone/renin ratio	2·57	1·42	
Corticosterone, nmol/mL	474.10	N/A	1.53-45.08
17-Hydroxyprogesterone, nmol/mL	0.8	N/A	2·0-8·0
Estradiol, ng/L	21	70	30·3-274·2
Testosterone, ng/mL	<0·10	<0·10	0·1-0·92
Dehydroepiandrosterone Sulfate	16·03	N/A	60·56-340·36
Androstenedione, nmol/L	<0·44	N/A	1·22-8·73
Progesterone, ng/mL	20·01	4·73	5·16-18·56
Follicle-Stimulating Hormone, mIU/mL	11·36	5·29	1·79-5·12
Luteinizing hormone, mIU/mL	17·96	5·02	1·2-12·86

N/A, Not available.

The constellation of clinical findings prompted genetic analysis which identified two variants in *CYP17A1*: c.1459_1467del (p.Asp487_Phe489del) and c.1226C>G (p.Pro409Arg). Parental testing showed a maternally inherited c.1459_1467del variant and a *de novo* c.1226C>G in the *CYP17A1*. These molecular findings, in conjunction with the clinical presentation, established the definitive diagnosis of 17-OHD.

### ​​Follow-up after therapeutic intervention​

Following treatment with hydrocortisone (10 mg once daily), dexamethasone (tapered from 0.375 mg daily to 0.375 mg every other day), spironolactone (20 mg twice daily), and estradiol-dydrogesterone, the patient achieved normalization of blood pressure and electrolyte levels, restoration of hormonal balance, and significant reduction in both ovarian cysts and adrenal hyperplasia (**Figures 2C, D**). Remarkably, follow-up breast MRI at six months demonstrated significant reduction in both mammary duct ectasia and the number/size of nodules (**Figures 1D–F**).

### Karyotype-based clinical comparisons

Notably, the systematic review identified 43 cases (including the present case) with near-complete breast development (Tanner stages 4–5), yet no cases of associated breast nodules or mammary duct ectasia were documented. Of these, karyotype data were available for 42 individuals: 29 (69.0%) were 46, XX, 12 were 46, XY (28.6%), and one had a mosaic karyotype of 47, XXX [3]/46, XX [47] which was included in the 46,XX in the following results (**Table 2**). The mean age of patients with 46,XX was significantly higher than that of 46,XY (29.5 ± 11.5 vs. 19.8 ± 6.9 years, *P* = 0.0095). There were no significant differences in height, weight, or BMI between the two groups. The prevalence of hypertension (62.5% vs. 33.3%) and hypokalemia (57.1% vs. 26.1%) were more frequently in patients with 46,XY though this difference was not statistically significant. Assessment of pubic hair Tanner staging revealed a significant difference between the two groups (*P* = 0.027). Among patients with 46,XX, 52.0% were at Tanner stage I and 12.0% reached stage V, whereas 46,XY individuals showed more advanced pubic hair development, with 37.5% reaching Tanner stage V and only 12.5% remaining at stage I.

**Table 2:** Clinical and demographic characteristics of patient with 17-OHD advanced breast development.

Characteristic	46,XX (n = 30)	46,XY (n = 12)	*P* value
Age, years (mean ± SD)	29.5 ± 11.5	19.8 ± 6.9	0.0095
Height, m (mean ± SD)	1.61 ± 0.05	1.68 ± 0.10	0.173
Weight, kg (mean ± SD)	58.9 ± 8.3	65.2 ± 18.4	0.406
Body mass index, kg/m² (mean ± SD)	22.9 ± 3.2	22.7 ± 4.1	0.899
Hypertension, n (%)	9 (33.3%)	5 (62.5%)	0.221
Hypokalemia, n (%)	6 (26.1%)	4 (57.1%)	0.181
Pubic Hair Tanner Stage, n (%)			0.027
- Stage I	13 (52.0%)	1 (12.5%)	
- Stage II	6 (24.0%)	2 (25.0%)	
- Stage III	1 (4.0%)	0 (0%)	
- Stage IV	2 (8.0%)	2 (25.0%)	
- Stage V	3 (12.0%)	3 (37.5%)	

*P* values were calculated using Student’s t-test or Chi-square test.

### Hormonal variations by sex chromosomes

To better understand the hormonal characteristics of patients with 17-OHD with breast development, we analyzed hormone levels across patients with 46,XX and 46,XY (**Table 3**). The majority of patients exhibited elevated progesterone, with 63.6% of 46,XX and 21.2% of 46,XY cases above the reference range. Estradiol levels showed a significant sex-based difference (*P* = 0.038), with 37.5% of patients with 46,XX below range, while most 46,XY were within or above normal. Androgen deficiency was common: testosterone was below the reference range in 51.7% of 46,XX and 27.6% of patients with 46,XY; similarly, dehydroepiandrosterone sulfate (DHEAS) and androstenedione were frequently reduced. Cortisol levels were subnormal in 31.3% of 46,XX and 15.6% of 46,XY. LH and FSH levels tended to be within or above range, suggesting hypergonadotropic hypogonadism, though no statistically significant differences were observed between karyotypes.

**Table 3:** Hormonal profiles of patients with 17-OHD with advanced breast development.

Hormones	Below range		Within range		Above range		*P* value
	46,XX	46,XY	46,XX	46,XY	46,XX	46,XY	
ACTH	0 (0)	0 (0)	5 (17.9)	1 (3.6)	19 (67.9)	3 (10.7)	1.000
LH	0 (0)	0 (0)	11 (29.7)	1 (2.7)	17 (45.9)	8 (21.6)	0.220
FSH	1 (2.7)	0 (0)	13 (35.1)	3 (8.1)	14 (37.8)	6 (16.2)	0.773
Cortisol	10 (31.3)	5 (15.6)	13 (40.6)	4 (12.5)	0 (0)	0 (0)	0.699
17-OHP	4 (12.5)	0 (0)	12 (37.5)	1 (3.1)	9 (28.1)	6 (18.8)	0.089
Estradiol	12 (37.5)	1 (3.1)	13 (40.6)	4 (12.5)	0 (0)	2 (6.3)	0.038
Progesterone	0 (0)	0 (0)	4 (12.1)	1 (3.0)	21 (63.6)	7 (21.2)	1.000
Testosterone	15 (51.7)	8 (27.6)	5 (17.2)	0 (0)	0 (0)	1 (3.4)	0.105
DHEAS	17 (63.0)	5 (18.5)	3 (11.1)	2 (7.4)	0 (0)	0 (0)	0.580
Androstenedione	14 (63.6)	4 (18.2)	3 (13.6)	1 (4.5)	0 (0)	0 (0)	1.000

ACTH, Adrenocorticotropic hormone; LH, Luteinizing hormone; FSH, Follicle-stimulating hormone; 17-OHP, 17-Hydroxyprogesterone; DHEAS, Dehydroepiandrosterone Sulfate. *P* values were calculated using Chi-square test. *P* values were calculated using Chi-square test.

### Variants distribution and genotype-phenotype analysis

**Figure 3:**
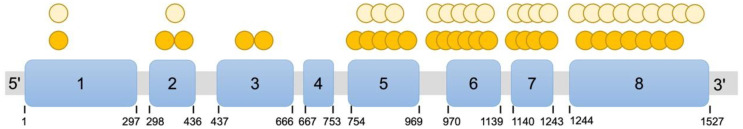
Distribution of *CYP17A1* (NM_000102) variants in patients with 17-OHD with advanced breast development. Blue rectangles​​ denote the eight exons of *CYP17A1*, with exon numbers and nucleotide positions indicated. Each orange circle represents one patient with a ​​homozygous variant​​ at that specific genomic coordinate. Each yellow circle represents one ​​allele with a heterozygous variant​​.

Of the 37 patients with available genetic data, those exhibiting breast development, which indicates residual enzymatic function, carried genotypes associated with partial loss of function. Only three patients harbored null variants, including a homozygous deletion of exons 1–6, p.Tyr329Lysfs*90, and p.Tyr329*. Among the remaining patients, 70.3% (26/37) were classified as C2 (homozygous or compound heterozygous non-null variants) and 29.7% (11/37) as NC (heterozygous null plus non-null variants). Mapping these variants to the *CYP17A1* gene revealed a predominant clustering in exons 5–8, with exon 8 representing a mutational hotspot (**Figure 3**). Genotype-phenotype correlation analysis showed no statistically significant differences in age, anthropometric measures, clinical characteristics, or hormone profiles between genotype groups (**Supplementary Tables S1**, **S2**).

## Discussion

The clinical manifestations of 17-OHD demonstrate significant heterogeneity, depending on varying degrees of residual enzymatic function associated with different sequencing variants. While breast underdevelopment has been well-documented in classical cases, we report for the first time a partial 17-OHD case presenting primarily with breast nodules and ductal ectasia. Notably, these mammary manifestations showed marked improvement following hormonal therapy, suggesting that appropriate diagnosis and endocrine management may prevent unnecessary surgical interventions in such patients.

As a rare subtype of CAH which have the prevalence of 1:14,000 to 1:18,000 in most populations (10, 11), 17-OHD demonstrates significant clinical heterogeneity due to varying degrees of residual enzymatic activity. Only 15–20% of these patients present with the classic triad of hypertension, hypokalemia, and disordered sexual development (12), making the diagnosis particularly challenging, especially in individuals with partial forms. Although breast underdevelopment is widely recognized as a typical feature of 17-OHD, previous reports indicate that approximately 6% (n = 445) of patients achieve advanced breast development, corresponding to Tanner stage 4-5 (3). The presence of breast development may lead to delayed diagnosis or misdiagnosis of 17-OHD. For instance, a 19-year-old patient with 46,XY, raised as a girl, was initially misdiagnosed with complete androgen insensitivity syndrome due to the presence of spontaneous Tanner stage 5 breast development (13), underscoring how atypical breast phenotypes can obscure the underlying diagnosis of 17-OHD.

In our analysis of the subset of the 17-OHD with advanced breast development, 69% were found to have a 46,XX karyotype, while 28.6% were 46,XY including four phenotypic males. Furthermore, we identified a rare case with mosaicism (47,XXX[3]/46,XX[47]) (14). These findings suggest that although estrogen deficiency is a typical feature of 17-OHD, a subset of patients may still attain near-complete secondary sexual development, regardless of chromosomal sex. Notably, the average diagnosis age of these patients was 26.9 years, with three individuals older than 50 years (14–16). Additionally, patients with 46,XX were diagnosed at a significantly older age than those with 46,XY. Individuals with 46,XY possibly present earlier due to more conspicuous phenotypes such as female external genitalia with absent secondary sexual development or primary amenorrhea, prompting earlier endocrine evaluation. In contrast, patients with 46,XX may exhibit milder or less specific symptoms such as menstrual irregularities or infertility, leading to delayed diagnosis (3). This age discrepancy likely reflects differences in the timing of clinical presentation and diagnosis between the two karyotype groups.

*CYP17A1* is primarily expressed in the adrenal cortex and gonads, but not in breast tissue, indicating that the breast development in patients with 17-OHD is secondary to the systemic hormonal changes (17). To better characterize the hormonal profiles of these patients, we analyzed available endocrine data and found that the majority (over 80%) exhibited low levels of androgens including testosterone, DHEAS and androstenedione, alongside elevated progesterone levels (85.3%). Estradiol levels were generally low to within the normal range, with 53.1% of these cases falling into normal range. Interestingly, although androgens are known precursors for estrogen biosynthesis, estradiol levels were within the normal range despite markedly reduced androgen levels. This observation suggests the existence of alternative or compensatory pathways for estrogen synthesis (18). It is possible that small amounts of androgen precursors such as DHEA or androstenedione are sufficient for downstream conversion, potentially facilitated by increased efficiency of local aromatase activity (19). Aromatase (cytochrome P450 19A1), the enzyme capable of converting C19 steroids into estrogens, is widely expressed in extragonadal tissues, including adipose tissue and brain (20). In fact, in prepubertal girls and postmenopausal women, extragonadal sites are the main sources of estrogens (21).

Estrogens play a central role in breast development (22) and also contribute to pubertal gynecomastia (20). However, estradiol level alone may not fully explain the degree of breast development observed, as some patients with 17-OHD with breast development still exhibited lower level of estradiol. It is also important to consider that “normal” estradiol levels may vary depending on institutional reference ranges. Furthermore, breast development is a complex, multi-hormonal process involving not only estrogens, but also growth hormone, insulin-like growth factor 1, and other regulators (22, 23), which warrant further investigation in the context of 17-OHD.

Interestingly, in our case, beyond the presence of developed breasts, the patient also presented with bilateral breast nodules and mammary duct ectasia, which showed significant clinical improvement after the hormonal therapy of 17-OHD. The development of these breast features likely results from a complex interplay of hormonal imbalances due to *CYP17A1* deficiency. Among the contributing factors, elevated progesterone is of particular interest, which was significantly decreased following hormonal therapy. Progesterone has been widely implicated in promoting breast cancer cell proliferation through multiple mechanisms, including activation of estrogen receptors, progesterone receptors, and G protein-coupled receptors such as GPR126 (24–26). The progesterone/RANKL signaling axis is recognized as a major regulator of breast epithelial proliferation (27), and progesterone antagonists can effectively inhibit progesterone-driven proliferation in mammary epithelial cells (25, 28). Supporting this association, a previous study identified a protein-truncating variant in *CYP17A1* in three sisters with early-onset breast cancer (29). Collectively, these findings underscore the importance of careful breast evaluation in patients with 17-OHD, not only for assessing secondary sexual development but also for the early detection of potential malignancies.

The vast majority of patients exhibiting breast development carried at least one non-null variant, strongly suggesting that partially retained 17,20-lyase activity is the key molecular driver for estrogen synthesis and subsequent initiation of puberty. Furthermore, variants were predominantly located in exons 5–8, with exon 8 being a particular hotspot. This region encodes the core catalytic domain of the enzyme, including the substrate-binding pocket and the crucial heme-binding region (8). This finding is consistent with previous studies which have identified sequencing variants in exons 6 and 8 as the prevalent variants in patients with 17-OHD (30). Moreover, missense variants like p.Arg347Cys and p.Arg449Cys, identified in this region, have been functionally characterized in previous studies (31). These sequencing variants severely impair but do not completely eliminate enzymatic activity (e.g., p.Arg347Cys retains 13.6% of 17α-hydroxylase activity (32)), which perfectly explains how they allow for partial sex hormone synthesis and lead to an incomplete clinical phenotype. In our case, the variants c.1459_1467del and c.1226C>G, located in exon 8 and exon 7 respectively, have both been documented as pathogenic (33). The in-frame deletion c.1459_1467del removes three amino acids (p.Asp487_Phe489del) and represents a common pathogenic variant in 17-OHD (4/52 alleles in a cohort of 26 patients), particularly in complete combined 17-OHD (33). Similarly, the c.1226C>G (p.Pro409Arg) variant has also been described in multiple cases (34, 35). In patients homozygous for c.1226C>G, phenotype severity varies by karyotype, with 46,XX showing milder manifestations, whereas a 46,XY case presented with a more severe phenotype (33, 34, 36).

Additionally, the low aldosterone in our case was confirmed through repeated testing at an independent third-party laboratory and a significantly elevated corticosterone level of 474.10 nmol/L (reference range: 1.53–45.08 nmol/L) was detected. As previously reported, the absence of 17-hydroxylase activity shunts steroidogenesis toward corticosterone instead of cortisol via 11-deoxycorticosterone, which is typically a minor adrenal product in humans but highly in rodent (1, 37). Consequently, the lack of cortisol synthesis in 17-OHD does not lead to true glucocorticoid deficiency because corticosterone excess compensates for glucocorticoid activity. Crucially, this excess 11-deoxycorticosterone and corticosterone strongly suppresses the renin–angiotensin–aldosterone system, resulting in the characteristic low-renin hypertension and profoundly suppressed aldosterone synthesis (38). Thus, the biochemical profile of our patient—low aldosterone, elevated corticosterone, hypertension, and hypokalemia—aligns closely with the established pathophysiology of 17-OHD.

## Conclusion

Due to its rarity and diverse clinical manifestations, 17-OHD is frequently overlooked or misdiagnosed in clinical practice. This study broadens the recognized phenotypic and hormonal spectrum of 17-OHD with advanced breast development, combined with low androgen levels, elevated progestogens, and hypergonadotropic hypogonadism, may serve as important diagnostic clues. Bilateral breast nodules and mammary duct ectasia may represent overlooked manifestations in patients with atypical breast development. Our findings emphasize the importance of increased clinical vigilance for these features, which may help avoid unnecessary surgical interventions and reduce the risk of delayed diagnosis or long-term oncologic complications.

## Data Availability

The original contributions presented in the study are included in the article/**Supplementary Material**. Further inquiries can be directed to the corresponding authors.

## References

[1] Auchus RJ. Steroid 17-hydroxylase and 17,20-lyase deficiencies, genetic and pharmacologic. J Steroid Biochem Mol Biol. (2017) 165:71–8. doi: 10.1016/j.jsbmb.2016.02.002, PMID: 26862015 PMC4976049

[2] Auer MK Nordenstrom A Lajic S Reisch N. Congenital adrenal hyperplasia. Lancet. (2023) 401:227–44. doi: 10.1016/S0140-6736(22)01330-7, PMID: 36502822

[3] Willemsen AL Torpy DJ De Sousa SMC Falhammar H Rushworth RL. 17alpha-hydroxylase/17,20-lyase deficiency (17-OHD): A meta-analysis of reported cases. J Clin Endocrinol Metab. (2025) 110:e1261–71. doi: 10.1210/clinem/dgae773, PMID: 39500362 PMC11913080

[4] Bolu S Eröz R Tekin M Doğan M. Atypical presentation in patients with 17 α-hydroxylase deficiency caused by a deletion in the CYP17A1 gene: short stature. Turk J Pediatr. (2020) 62:851–7. doi: 10.24953/turkjped.2020.05.019, PMID: 33108090

[5] Batatinha JAP Nishi MY Batista RL Faria Júnior JAD Sircili MHP Denes FT. Estrogen-secreting testicular tumors in 46,XY female patients with 17α-hydroxylase/17,20-lyase deficiency: two unusual case reports and a review of the literature. Front Genet. (2025) 16:1508792. doi: 10.3389/fgene.2025.1508792, PMID: 40313596 PMC12043694

[6] Brooke AM Taylor NF Shepherd JH Gore ME Ahmad T Lin L. A novel point mutation in P450c17 (CYP17) causing combined 17alpha-hydroxylase/17,20-lyase deficiency. J Clin Endocrinol Metab. (2006) 91:2428–31. doi: 10.1210/jc.2005-2653, PMID: 16569739

[7] İsakoca M Erdeve Ş Çetinkaya S. Rare types of congenital adrenal hyperplasias other than 21-hydroxylase deficiency. J Clin Res Pediatr Endocrinol. (2025) 17:23–32. doi: 10.4274/jcrpe.galenos.2024.2024-6-21-S, PMID: 39713884 PMC11730102

[8] Zhang D Yao F Luo M Wang Y Tian T Deng S. Clinical characteristics and molecular etiology of partial 17alpha-hydroxylase deficiency diagnosed in 46,XX patients. Front Endocrinol (Lausanne). (2022) 13:978026. doi: 10.3389/fendo.2022.978026, PMID: 36589849 PMC9797673

[9] He QB Wu CH Sun DL Yuan JY Hu HY Yang K. Functional assessment of a novel biallelic MYH3 variation causing CPSKF1B (contractures, pterygia, and spondylocarpotarsal fusion syndrome1B). Mol Genet Genomic Med. (2024) 12:e2401. doi: 10.1002/mgg3.2401, PMID: 38444278 PMC10915484

[10] Speiser PW Arlt W Auchus RJ Baskin LS Conway GS Merke DP. Congenital adrenal hyperplasia due to steroid 21-hydroxylase deficiency: an endocrine society clinical practice guideline. J Clin Endocrinol Metab. (2018) 103:4043–88. doi: 10.1210/jc.2018-01865, PMID: 30272171 PMC6456929

[11] Xia J Liu F Wu J Xia Y Zhao Z Zhao Y. Clinical and genetic characteristics of 17 α-hydroxylase/17, 20-lyase deficiency: c.985_987delTACinsAA mutation of CYP17A1 prevalent in the chinese han population. Endocr Pract. (2021) 27:137–45. doi: 10.4158/EP-2020-0478, PMID: 33547012

[12] Fontenele R Costa-Santos M Kater CE. 17α-hydroxylase deficiency is an underdiagnosed disease: high frequency of misdiagnoses in a large cohort of Brazilian patients. Endocr Pract. (2018) 24:170–8. doi: 10.4158/EP171987.OR, PMID: 29144824

[13] Sarathi V Reddy R Atluri S Shivaprasad C. A challenging case of primary amenorrhoea. BMJ Case Rep. (2018) 2018. doi: 10.1136/bcr-2018-225447, PMID: 30002216 PMC6047692

[14] Yao JT Xu MZ Zhang YR Wang BR Li MR Gao L. Refractory hypokalemia with sexual dysplasia and infertility caused by 17α-hydroxylase deficiency and triple X syndrome: A case report. Open Life Sci. (2023) 18:20220548. doi: 10.1515/biol-2022-0548, PMID: 36820210 PMC9938537

[15] Yamagata S Kageyama K Usui T Saito K Takayasu S Usutani M. Identification of a homozygous c.1039C>T (p.R347C) variant in CYP17A1 in a 67-year-old female patient with partial 17α-hydroxylase/17,20-lyase deficiency. Endocr J. (2022) 69:115–20. doi: 10.1507/endocrj.EJ21-0266, PMID: 34483146

[16] Ueda Y Usui T Watanabe T Kaneko K Nakatani R Kakita-Kobayashi M. Elevated levels of plasma immunoassayable aldosterone in a mild form of 17 alpha-hydroxylase/17,20-lyase deficiency diagnosed at the age of 50. AACE Clin Case Rep. (2015) 1:e156–60. doi: 10.4158/EP14388.CR

[17] Mak PJ Duggal R Denisov IG Gregory MC Sligar SG Kincaid JR. Human cytochrome CYP17A1: the structural basis for compromised lyase activity with 17-hydroxyprogesterone. J Am Chem Soc. (2018) 140:7324–31. doi: 10.1021/jacs.8b03901, PMID: 29758981 PMC5999583

[18] Ishikawa T Glidewell-Kenney C Jameson JL. Aromatase-independent testosterone conversion into estrogenic steroids is inhibited by a 5 alpha-reductase inhibitor. J Steroid Biochem Mol Biol. (2006) 98:133–8. doi: 10.1016/j.jsbmb.2005.09.004, PMID: 16386416

[19] Liu T Huang Y Lin H. Estrogen disorders: Interpreting the abnormal regulation of aromatase in granulosa cells (Review). Int J Mol Med. (2021) 47:73. doi: 10.3892/ijmm.2021.4906, PMID: 33693952 PMC7952251

[20] Shi Z Xin M. Endocrine hormones and their impact on pubertal gynecomastia. J Clin Med. (2024) 14:158. doi: 10.3390/jcm14010158, PMID: 39797240 PMC11721017

[21] Cui J Shen Y Li R. Estrogen synthesis and signaling pathways during aging: from periphery to brain. Trends Mol Med. (2013) 19:197–209. doi: 10.1016/j.molmed.2012.12.007, PMID: 23348042 PMC3595330

[22] Hannan FM Elajnaf T Vandenberg LN Kennedy SH Thakker RV. Hormonal regulation of mammary gland development and lactation. Nat Rev Endocrinol. (2023) 19:46–61. doi: 10.1038/s41574-022-00742-y, PMID: 36192506

[23] Kleinberg DL Wood TL Furth PA Lee AV. Growth hormone and insulin-like growth factor-I in the transition from normal mammary development to preneoplastic mammary lesions. Endocr Rev. (2009) 30:51–74. doi: 10.1210/er.2008-0022, PMID: 19075184 PMC5393153

[24] An W Lin H Ma L Zhang C Zheng Y Cheng Q. Progesterone activates GPR126 to promote breast cancer development via the Gi pathway. Proc Natl Acad Sci U.S.A. (2022) 119:e2117004119. doi: 10.1073/pnas.2117004119, PMID: 35394864 PMC9169622

[25] Trabert B Sherman ME Kannan N Stanczyk FZ. Progesterone and breast cancer. Endocr Rev. (2020) 41:320–44. doi: 10.1210/endrev/bnz001, PMID: 31512725 PMC7156851

[26] Deng Y Jin H. Effects of menopausal hormone therapy-based on the role of estrogens, progestogens, and their metabolites in proliferation of breast cancer cells. Cancer Biol Med. (2021) 19:432–49. doi: 10.20892/j.issn.2095-3941.2021.0344, PMID: 34779589 PMC9088189

[27] Tanos T Sflomos G Echeverria PC Ayyanan A Gutierrez M Delaloye JF. Progesterone/RANKL is a major regulatory axis in the human breast. Sci Transl Med. (2013) 5:182ra55. doi: 10.1126/scitranslmed.3005654, PMID: 23616122

[28] Northey JJ Hayward MK Yui Y Stashko C Kai F Mouw JK. Mechanosensitive hormone signaling promotes mammary progenitor expansion and breast cancer risk. Cell Stem Cell. (2024) 31:106–126.e13. doi: 10.1016/j.stem.2023.12.002, PMID: 38181747 PMC11050720

[29] Hopper JL Hayes VM Spurdle AB Chenevix-Trench G Jenkins MA Milne RL. A protein-truncating mutation in CYP17A1 in three sisters with early-onset breast cancer. Hum Mutat. (2005) 26:298–302. doi: 10.1002/humu.20237, PMID: 16121340

[30] Zhang M Sun S Liu Y Zhang H Jiao Y Wang W. New, recurrent, and prevalent mutations: Clinical and molecular characterization of 26 Chinese patients with 17alpha-hydroxylase/17,20-lyase deficiency. J Steroid Biochem Mol Biol. (2015) 150:11–6. doi: 10.1016/j.jsbmb.2015.02.007, PMID: 25697092

[31] Auchus RJ Miller WL. Molecular modeling of human P450c17 (17alpha-hydroxylase/17,20-lyase): insights into reaction mechanisms and effects of mutations. Mol Endocrinol. (1999) 13:1169–82. doi: 10.1210/mend.13.7.0326, PMID: 10406467

[32] Van Den Akker EL Koper JW Boehmer AL Themmen AP Verhoef-Post M Timmerman MA. Differential inhibition of 17alpha-hydroxylase and 17,20-lyase activities by three novel missense CYP17 mutations identified in patients with P450c17 deficiency. J Clin Endocrinol Metab. (2002) 87:5714–21. doi: 10.1210/jc.2001-011880, PMID: 12466376

[33] Cao Y Zhao Z Lu L Zhang X Zhang W Sun B. CYP17A1 pathogenic variants in 26 Chinese patients with 17α-hydroxylase deficiency by targeted long-read sequencing. J Clin Endocrinol Metab. (2024) 110:59–69. doi: 10.1210/clinem/dgae414, PMID: 38934795

[34] Pan P Zheng L Huang J Chen X Ni R Zhang Q. Endocrine profiles and cycle characteristics of infertile 17α-hydroxylase/17,20-lyase Deficiency Patients undergoing assisted Reproduction Treatment: a retrospective cohort study. J Ovarian Res. (2023) 16:111. doi: 10.1186/s13048-023-01190-6, PMID: 37316894 PMC10265862

[35] Shi M Chen X Zhou Q Shen F. Clinical and genetic analyses of a Chinese female with 17α-hydroxylase/17,20-lyase deficiency. Gynecol Endocrinol. (2014) 30:890–3. doi: 10.3109/09513590.2014.943721, PMID: 25027547

[36] Lam CW Arlt W Chan CK Honour JW Lin CJ Tong SF. Mutation of proline 409 to arginine in the meander region of cytochrome p450c17 causes severe 17 alpha-hydroxylase deficiency. Mol Genet Metab. (2001) 72:254–9. doi: 10.1006/mgme.2000.3134, PMID: 11243732

[37] Tiosano D Knopf C Koren I Levanon N Hartmann MF Hochberg Z. Metabolic evidence for impaired 17alpha-hydroxylase activity in a kindred bearing the E305G mutation for isolate 17,20-lyase activity. Eur J Endocrinol. (2008) 158:385–92. doi: 10.1530/EJE-07-0712, PMID: 18299473

[38] Morris DJ Latif SA Brem AS. An alternative explanation of hypertension associated with 17α-hydroxylase deficiency syndrome. Steroids. (2014) 79:44–8. doi: 10.1016/j.steroids.2013.10.006, PMID: 24176792

