# Supplementary material for: Bilateral breast nodules as an unusual manifestation of 17α-hydroxylase/17,20-lyase deficiency
Source: Front Endocrinol (Lausanne). 2025 Oct 27;16:1658362. doi: 10.3389/fendo.2025.1658362 (PMC12597753; doi:10.3389/fendo.2025.1658362)
Supplement: Supplementary file 1 [file DataSheet1.pdf]

Table S1. Characteristics of 17-OHD Patients with Advanced Breast Development across Genotypes.

Characteristic	C2 (n = 26)	CN (n = 11)	P value
Age, years (mean ± SD)	26.1 ± 13.3	27.5 ± 10.6	0.763
Height, m (mean ± SD)	1.63 ± 0.08	1.63 ± 0.07	0.948
Weight, kg (mean ± SD)	60.4 ± 12.0	58.1 ± 9.3	0.628
BMI, kg/m^2 (mean ± SD)	22.8 ± 3.1	21.8 ± 3.4	0.448
Hypertension, n (%)	8 (38.1%)	4 (44.4%)	1.000
Hypokalemia, n (%)	4 (25.0%)	4 (44.4%)	0.394
Pubic Hair Tanner Stage, n (%)			0.717
- Stage I	10 (45.5%)	3 (33.3%)	
- Stage II	4 (18.2%)	3 (33.3%)	
- Stage III	1 (4.5%)	0 (0%)	
- Stage IV	2 (9.1%)	2 (22.2%)	
- Stage V	5 (22.7%)	1 (11.1%)	

P values were calculated using Student's t-test or Chi-square test.

C2: two non-null variants; NC: one null and one non-null variant.

Table S2 Hormonal Profiles of Patients with 17-OHD with Advanced Breast Development across Genotypes.

Hormones	Below Range		Within Range		Above Range		P value
	C2	CN	C2	CN	C2	CN	
ACTH	0 (0)	0 (0)	4 (16.0)	2 (8.0)	12 (48.0)	7 (28.0)	1.000
LH	0 (0)	0 (0)	8 (25.0)	4 (12.5)	14 (43.8)	6 (18.8)	1.000
FSH	1 (3.1)	0 (0)	10 (31.3)	6 (18.8)	11 (34.4)	4 (12.5)	0.797
Cortisol	9 (30.0)	6 (20.0)	10 (33.3)	5 (16.7)	0 (0)	0 (0)	1.000
17-OHP	2 (7.4)	1 (3.7)	9 (33.3)	4 (14.8)	9 (33.3)	2 (7.4)	0.841
Estradiol	7 (25.9)	2 (7.4)	9 (33.3)	7 (25.9)	2 (7.4)	0 (0)	0.362
Progesterone	0 (0)	0 (0)	4 (13.3)	16 (53.3)	1 (3.3)	9 (30.0)	0.640
Testosterone	11 (44.0)	8 (32.0)	4 (16.0)	1 (4.0)	1 (4.0)	0 (0)	0.753
DHEAS	13 (50.0)	8 (30.8)	3 (11.5)	2 (7.7)	0 (0)	0 (0)	1.000
Androstenedione	13 (59.1)	5 (22.7)	1 (4.5)	3 (13.6)	0 (0)	0 (0)	0.117

ACTH, Adrenocorticotropic hormone; LH, Luteinizing hormone; FSH, Follicle-stimulating hormone; 17-OHP, 17-Hydroxyprogesterone; DHEA-S, Dehydroepiandrosterone Sulfate. P values were calculated using Chi-square test.

Figure S1 Physical examination and histopathologic characteristics of sexual characteristics.

(A-B) Physical examination shows Tanner 4 breast development (A), naïve vulva and absent of pubic hair (B).

(C-D) Histopathological analysis of a breast nodule obtained prior to the diagnosis of 17α-hydroxylase/17,20-Lyase deficiency (17-OHD) shows ductal papilloma (C) and dilated breast ducts (D). Haematoxylin and eosin stain. Scale bars represent 40 μm.

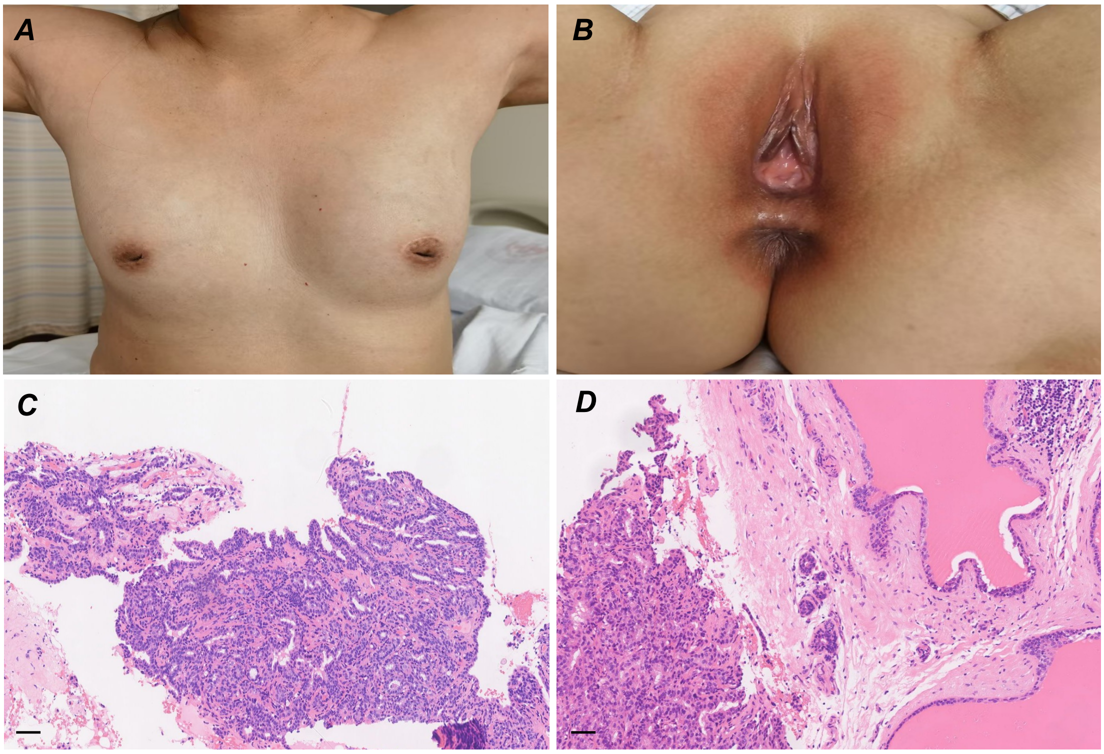
A
B
C
D
